# Do elderly patients benefit from implantable-cardioverter defibrillators?

**DOI:** 10.1007/s12471-014-0559-3

**Published:** 2014-05-08

**Authors:** J. R. de Groot

**Affiliations:** Heart Center, Department of Cardiology, Academic Medical Center/University of Amsterdam, Meibergdreef 9, 1105 AZ Amsterdam, the Netherlands

Sudden cardiac death resulting from lethal ventricular arrhythmias forms a major cause of mortality. The implantable cardioverter defibrillator (ICD) has been shown to reduce mortality in patients who survived cardiac arrest, who experienced a previous myocardial infarction and have a diminished left ventricular ejection fraction (LVEF), or who have a dilated cardiomyopathy and an LVEF. This has been demonstrated and verified in several randomised trials, and has subsequently entered the guidelines (1). Consequently, the implantation rate of ICDs has increased exponentially worldwide over the last decade.

A case can be made that this conveys an enormous success of medical technology to improve quality and quantity of life. Indeed, it was recently reported that there were fewer cases of ventricular fibrillation encountered during out-of-hospital cardiac arrest between 2005–2008, compared with the time frame 1995–1997, which could be explained in part (33 %) by the increased number of ICD carriers among subjects vulnerable for sudden death.(2) However, ICDs only protect against ventricular arrhythmia-induced cardiac arrest, and the question arises whether some ICD candidates may not have too many life-threatening comorbid conditions to have the chance to experience gain in life expectancy from ICD implantation.

Therefore, Goldenberg et al. performed a post hoc analysis of the MADIT 2 trial, and proposed a simple and easily applicable clinical model of five risk factors associated with the usefulness of ICD therapy.(3;4) They showed that a risk score model including: 1) age above 70 years, 2) renal insufficiency (defined as blood urea nitrogen >26 mg/dl), 3) atrial fibrillation, 4) NYHA class >2 and 5) a QRS complex >120 msec on the ECG was able to predict clinical benefit of ICD implantation. In their analysis, ICD implantation was futile in patients with none of these risk factors, as no events urging ICD therapy occurred. The curve of ICD benefit was U shaped, however, and on its right side were patients with 3 or more risk factors. These patients had no benefit from ICD therapy, because they died from other than arrhythmic causes such as advanced heart failure. Hence, optimal benefit from the ICD was gained in the patient group with more than 0 but less than 3 risk factors. It is suggested that within the population of patients conforming to the inclusion criteria of the randomised ICD trials, or conforming to the guidelines, patient selection may potentially be further optimised to select those patients who will really experience net benefit from the ICD. Despite the elegance and simplicity of their model, the criteria proposed by Goldenberg et al. have not found their way into the guidelines yet. One of the possible explanations for this is that the clinical decision to abstain from ICD therapy can be difficult, and that ICDs are readily implanted when the patient’s life expectancy is estimated to be more than 1 year. Estimating life expectancy may be complex at the bedside, but a very similar set of risk factors, namely age ≥75 years, estimated glomerular filtration rate (eGFR) ≤30 ml/min/1.73 m^2^ and LVEF ≤20 % was recently reported to be associated with 2.5, 13.2 and 46.3 % mortality within the first year after primary prevention ICD implantation for ≤1, 2 and 3 risk factors respectively.(3) Another explanation could be that many patients eligible for ICD therapy are beyond the age of 75 at the time the indication is set, which would, according to the Goldenberg criteria, directly put them at least into the intermediate risk category, and seemingly prevent the need for further risk stratification. Given the fact that many ICD recipients are old, the question whether advanced age is a (relative) contraindication for ICD implantation is eminent, but the efficacy of ICD therapy in the elderly remains largely unknown.

In this issue of the *Netherlands Heart Journal*, Anné et al. investigated the validity of the Goldenberg risk factors (QRS >120 msec, NYHA class >2, atrial fibrillation, defined as at least one documented episode, and renal failure, defined as eGFR <60 ml/min/1.73 m^2^) in patients of 75 years or older, evidently with exclusion of age as a risk criterion.(4) They performed a retrospective analysis of the ICD database of two large implantation centres: the Erasmus MC and the University Hospital in Basel, Switzerland, and selected 179 patients who were 75 years or older at the time of ICD implantation. Furthermore, they compared survival in these elderly ICD carriers with a sample of ICD patients of 60–70 years from the same database, and with the general population older than 75 years, as derived from the mortality statistics of the Dutch national statistics agency (Centraal Bureau voor de Statistiek, CBS).

Within the group of 179 elderly ICD carriers, patients with 2 or more risk factors had a worse prognosis than those with 0 or 1 risk factor. Median survival was 6.2, 4.2 and 3.5 years in elderly patients with 0, 1 and ≥2 risk factors respectively. Furthermore, the entire group of elderly patients did worse than a random sample of ICD carriers aged 60–70 years, derived from the same databases. This may not sound surprising, albeit that the survival curves started to deviate not earlier than 3 years after implantation. Interestingly, elderly ICD patients with 0 risk factors had the same mortality risk as the age-matched general Dutch population.

Within the group of elderly ICD carriers, there was no significant relation between the number of risk factors and the occurrence of appropriate ICD shocks; on the other hand, patients who did not experience appropriate therapy were better off, as mortality in that group was significantly lower than in the group with appropriate therapy.

Of the elderly patients who died during the observation period, 49 % died without ever having experienced appropriate ICD therapy. This number was 29 % in the patients with no risk factors, and amounted to 64 % in those with two or more risk factors, underlining the notion that patients with heavy comorbidity die from causes other than lethal arrhythmias, and that ICD implantation may potentially be futile in those patients.

There are, as Anné and coworkers do acknowledge and discuss, several remarks to be made about the study. First, this was a retrospective analysis of patients who were implanted with an ICD, hence there is an obvious selection bias, as patients who refused or were denied ICD implantation were not included (which is similar to the ad hoc analysis of the MADIT 2 trial in the Goldenberg paper (5)). Therefore, it cannot be excluded that elderly ICD patients with 2 or more risk factors and a poor prognosis still performed better than similar patients without an ICD. Second, the number of patients is relatively low, and follow-up was incomplete and had to be censored for 22 patients (12 %). This evidently puts a confidence interval on the interpretation of the data. Third, due to expanded indications for primary prevention implantation after the publication of the MADIT 2 and SCD-HeFT trial in 2002 and 2005 respectively (6, 7), the mean follow-up for patients with a primary prevention indication was shorter than that of patients with a secondary prevention indication, as data collection for this study already started in 1999. This may also have introduced bias, as the patients with the longer follow-up may be overrepresented in the mortality numbers.

Despite this, and taking into account that ICD harm, in the form of inappropriate shocks, infection and implantation-related complications, was not included in this analysis, the data presented by Anné and coworkers are of considerable importance for patients, clinicians and policymakers. They do show, namely, that advanced age per se is not a criterion that should affect the decision to implant an ICD. In the era of extensive guidelines, consensus papers, decision rules, and a limited health care budget, the decision to implant an ICD still comes down to an individual interaction between doctor and patient, in which the patient’s comorbidity, expectations and conceptions need to be taken into account. Anné et al. provide us with the necessary numbers to be able to manage these expectations more appropriately, and to improve the care of patients with an increased risk of (sudden arrhythmic) death.
